# Recto-Anal Junction (RAJ) and Fecal Microbiomes of Cattle Experimentally Challenged With *Escherichia coli* O157:H7

**DOI:** 10.3389/fmicb.2020.00693

**Published:** 2020-04-17

**Authors:** Raies A. Mir, Robert G. Schaut, Torey Looft, Heather K. Allen, Vijay K. Sharma, Indira T. Kudva

**Affiliations:** ^1^Food Safety and Enteric Pathogens Research Unit, National Animal Disease Center, Agricultural Research Service, United States Department of Agriculture, Ames, IA, United States; ^2^ARS Research Participation Program, Oak Ridge Institute for Science and Education (ORISE), Oak Ridge, TN, United States

**Keywords:** cattle, recto-anal junction, O157, colonization, microbiota

## Abstract

Cattle are the asymptomatic reservoirs of *Escherichia coli* O157:H7 (O157) that preferentially colonizes the bovine recto-anal junction (RAJ). Understanding the influence of O157 on the diversity of the RAJ microbiota could give insights into its persistence at the RAJ in cattle. Hence, we compared changes in bovine RAJ and fecal microbiota following O157 challenge under experimental conditions. Cattle were either orally challenged (*n* = 4) with10^10^ CFU of a streptomycin-resistant O157 strain 86–24, or mock-challenged (*n* = 4) with phosphate buffered saline. Rectoanal mucosal swab (RAMS) and fecal samples were collected at different time points for analysis. Alpha diversity measures (Chao1 species richness and Shannon diversity index) were found to be significantly different between RAMS and fecal samples but not influenced by O157 challenge. The *Firmicutes* to *Bacteroidetes* (F: B) ratio was higher in RAMS samples from O157 colonized animals and this may have influenced the consistent yet decreased O157 colonization at the RAJ. Specific bacterial genera that were present in relative low abundance in fecal and RAMS microbiota did not affect overall microbial diversity but were associated with O157 colonization. Differential abundance analysis (DAA) of genera in samples from O157 shedding cattle indicated significantly higher relative abundance of *Paenibacillus* and *Fusobacterium* in RAMS, and *Tyzzerella* in fecal samples. Mock-challenged cattle showed higher relative abundance of *Intestinimonas* and *Citrobacter* in RAMS samples, and *Succinivibrio*, and *Prevotella 1* in fecal samples. These results suggest that O157 challenge exerts transient influence on the intestinal microbial community which in turn might promote O157 colonization in a site-specific manner.

## Introduction

Shiga toxin-producing *Escherichia coli* (STEC) cause 265,000 illnesses in the United States (Scallan et al., 2011) and 2.8 million infections globally (Majowicz et al., 2014); 36% of these illnesses are attributed to STEC O157:H7 (O157) (Davis et al., 2005). In addition, a combined economic loss to public health, agriculture and meat industry estimated at $993 million per year has been attributed to STEC (mainly O157) contamination of foods and human infections (Hoffmann et al., 2012; Scharff, 2012). O157 colonizes the gastrointestinal tract (GIT) of cattle asymptomatically, but causes bloody diarrhea, hemorrhagic colitis (HC), and hemolytic uremic syndrome (HUS) in humans (Riley et al., 1983; Davis et al., 2014). Contamination of foods with bovine feces is a major risk factor for human infection and the preferential colonization of O157 at the bovine rectoanal junction (RAJ) contributes toward increased O157 load in bovine feces (Mechie et al., 1997; Elder et al., 2000; Naylor et al., 2003). Histologically, RAJ transitions along its length from columnar epithelium that lines the rectum (rich in lymphoid tissue, secretory, and absorptive functions) to stratified squamous epithelium lining the anus (often keratinized to protect underlying tissues) (Lim et al., 2007; Kudva and Stasko, 2013). The mechanism of O157 tropism for RAJ is not fully understood, nevertheless, RAJ is the main source of fecal O157 which is rarely attributed to transient passage through the proximal sections of GIT in cattle (Naylor et al., 2003; Fox et al., 2008). In fact, swabbing of RAJ using a foam-tipped applicator (rectoanal mucosal swab or RAMS) is a sensitive sampling method for detecting O157 in cattle (Rice et al., 2003).

Analysis of the structural and functional profiles of cattle GIT microbiota has gained pace recently (Mao et al., 2015), especially in the context of the rumen microbiome and feed utilization by cattle (Callaway et al., 2010; Petri et al., 2013; Myer et al., 2017; Thomas et al., 2017). In addition, reports have established a link between younger animals and GIT microbiota with Shiga toxin-producing *E. coli* (STEC) shedding (Mir et al., 2015, 2016, 2019). For example, Shannon diversity index was reported to be higher in fecal samples from non-shedding animals and lower in feces with increasing STEC concentration (Mir et al., 2016). Likewise, GIT microbiota has also been shown to play a role in O157 shedding by cattle as evidenced by the heterogeneity in fecal microbiota diversity between non-shedding and super-shedder cattle (Xu et al., 2014).

Microbiota composition, species richness, and Shannon diversity index differ between various GIT regions, particularly in the forestomach versus intestines (Mao et al., 2015). A study analyzing tissue and digesta samples at the time of slaughter from steers identified as super-shedders (*n* = 5) and non-shedding (*n* = 5), showed higher number of operational taxonomic units (OTUs) and species richness in spiral colon and lower GIT of super-shedding cattle (Zaheer et al., 2017). However, when all samples from upper and lower GIT were combined in the analysis, the samples did not cluster by shedding status mainly due to the differences in microbiota among various regions of GIT (Zaheer et al., 2017). In one study, the 25 most common genera accounted for more than 85% of ruminal and fecal bacterial populations (Callaway et al., 2010), and in another study, 45 OTUs in rectal samples were shared by all animals (Zaheer et al., 2017).

Changes in GIT microbiota can occur due to dietary change (Myer et al., 2017), infection, or immune system failure (Ichinohe et al., 2011; Wu and Wu, 2012). In cattle under certain conditions, O157 can cause inflammation, small mucosal hemorrhages in intestine, and induce immune responses (Walle et al., 2013), suggesting that it could also be disturbing GIT microbiota during its passage. Also, O157 isolates from colon contents and fecal swabs showed similar pulsed-field gel electrophoresis (PFGE) patterns, but differed from O157 isolated from RAMS and submucosal swabs in cattle (Fox et al., 2008), indicating that transient (passing through GIT), and colonized (adhered to tissues) O157 may behave differently and may effect varying changes in the GIT versus RAJ microbiota.

A recent study suggested that differences in compositional and functional levels of microbiota in RAJ tissue samples may be associated with O157 super-shedding in cattle (Wang et al., 2018). The study analyzed RAJ tissue samples collected at slaughter from steers previously identified as super-shedders (*n* = 5) and non-shedding (*n* = 4) based on the levels of O157 in fecal samples before slaughter (Wang et al., 2018). Comparison between super-shedders and non-shedding cattle indicated no significant difference in Chao1 species richness and Shannon diversity index at the RAJ (Wang et al., 2018). Although the alpha indices were similar between the two groups of cattle, there were differences in the composition of the RAJ-associated microbiota. At the phylum level, nine phyla were detected in both super-shedders and non-shedding cattle, while four phyla, including *Elusimicrobia*, *Fusobacteria*, *Lentisphaerae*, and OD1 were detected only in non-shedding cattle (Wang et al., 2018). OTU45 and OTU180, closely related to *Paludibacter*, were more abundant in non-shedding cattle but OTU56, closely related to *Bacteroides*, and OTU121 closely related to *Clostridium*, were more abundant in super-shedder cattle (Wang et al., 2018).

It is hard to replicate microbiota studies due to high degree of variability among individual samples, animal to animal variation, and variation due to sample processing. For example, *Ruminococcus* was reported to be abundant in O157 super-shedding steers, but further analysis indicated that it was due to only one super-shedding animal (Zaheer et al., 2017). A higher abundance of *Prevotella* was observed among non-shedders in that study (Zaheer et al., 2017) but in another study, *Alistipes* and *Prevotella* were found to be more abundant in O157 super-shedding cattle (Xu et al., 2014). Also, animal-to-animal variations in cattle fecal and RAMS microbiota with respect to taxonomic profiles, beta-diversity analyses, and the relative abundances of shared taxa (taxa present in all the animals) were reported, suggesting that host animal is the main drive for these bacterial community structures (Durso et al., 2010; Wang et al., 2018).

Based on these reports, we hypothesized that the inoculation of cattle with O157 would cause changes in the GIT microbiota which would differ between RAMS and fecal samples after colonization. We evaluated this hypothesis by comparing the RAMS and fecal bacterial community structure, before and after O157 challenge, under controlled experimental conditions to minimize the variabilities of a field study. This is a first of its kind study comparing the changes in, and association of, RAJ and fecal microbiota with O157 colonization in experimentally challenged cattle. Our results suggest that O157 challenge exerts transient influence on the intestinal microbial community which in turn might promote O157 colonization in a site-specific manner.

## Materials and Methods

### Animal Management

Standard practices of husbandry and veterinary care were applied to animals used in this study. The research protocols used were approved by the USDA-ARS-Institutional Animal Care and Use Committee. In the pilot study, four Holstein steers (∼1 year old), maintained at the National Animal Disease Center (NADC, Ames, IA, United States) were sampled to verify if there were inherent differences in the taxonomic profiles and diversity of microbiota in the fecal and recto-anal mucosal swabs (RAMS) samples and to verify if our sampling method would capture these differences. In the main study, Jersey steers (6–8 months old) (*n* = 8) were tagged with a unique identification number and randomly assigned to one of the following two groups: (1) Challenged (Chx; *n* = 4) and (2) Mock-challenged (Mck; *n* = 4). Microbiota from the fecal and RAMS samples were compared before and after O157 or mock challenge of these cattle. All the steers were fed pelleted feed (Kent Calf Creep^TM^, Kent Nutrition Group, Inc., Muscatine, IA, United States), alfalfa hay and *ad libitum* water with limited access to pasture, when housed outside in field barns. A day before the challenge (Day -1), the two groups of Jersey steers were moved inside into separate rooms in a climate controlled BSL2 facility at NADC and continued to be fed pelleted feed (Kent Calf Creep^TM^) and alfalfa hay cubes (Ontario Dehy Inc., Goderich, ON, Canada) in amounts equal to 1% of their body weights, twice daily, with *ad libitum* water.

### Oral Challenge With O157 and Sample Collection

**FIGURE 1:**
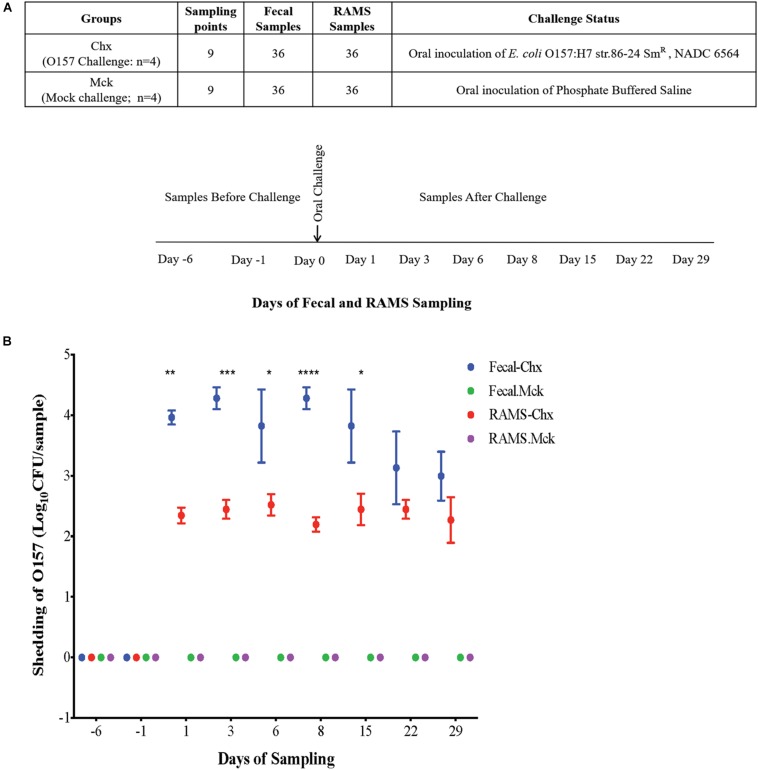
**(A)** Experimental design and sampling strategy used in this study. Steers were randomly assigned to two groups on Day -6. Challenged (Chx) steers (*n* = 4) were orally inoculated with O157 on Day 0 while the mock challenged (Mck) steers (*n* = 4) received sterile phosphate buffered saline. Fecal and RAMS samples for microbiota analysis were collected at Days -6, -1 before challenge, and Days 1, 3, 6, 8, 15, 22, and 29 after challenge. **(B)** O157 culture results of fecal and RAMS samples collected from steers before and after challenge. Counts are shown as Log_10_ CFU/200 mg sample. *p < 0.05, **p < 0.01, ***p < 0.001, and ****p < 0.0001.

All the Jersey steers, in the main study, were moved into the BSL2 facility 1 day before oral challenge (Day -1) with O157. On Day 0, four steers in one room were orally challenged with 10^10^ colony forming units (CFUs) of a sequenced, curli-negative isolate (NADC 6564; Accession # CP017251) derived from the streptomycin-resistant O157 strain 86–24 (86–24 Sm^*R*^, Stx2+) (Challenged or Chx group) (Sharma et al., 2016) and four steers in another room were mock-challenged with 10 mL of sterile phosphate-buffered saline (PBS) (Mock challenged or Mck) (Figure 1A). Fecal and RAMS samples were collected from all animals at Day -6 when all animals were still outside on a pasture, and after moving animals indoors on Days -1, 1, 3, 6, 8, 15, 22, and 29, resulting in 72 fecal and 72 RAMS to a combined total of 144 samples. Fecal samples (∼10 grams) were collected directly from the animal by rectal palpation, and RAMS samples were collected by swabbing the recto-anal junction (RAJ) area with four sterile foam-tipped applicators swabs (Thermo Scientific, Rockford, IL, United States) after the RAJ mucosa was thoroughly swabbed to clear any visible feces. Fecal and RAMS samples were put in separate sterile tubes and transported on ice to the laboratory on the same day. Pre-challenge samples were collected on Days -6, and -1 and all animals were challenged on Day 0 (Figure 1A). Post-challenge sampling was done on Days 1, 3, 6, 8, 15, 22, and 29 (Figure 1A).

### Detection and Enumeration of O157 Challenge Strain NADC 6564

Ten grams of feces and four RAMS swabs per animal were suspended in separate 50 ml trypticase soy broth (TSB; Difco-Becton Dickenson, Sparks, MD, United States) flasks; approximately 100 mg of mucosal sample was collected on each RAMS swab. TSB suspensions were plated directly or after overnight enrichment at 37°C as described previously (Mir et al., 2019). Plates with sorbitol-MacConkey (SMAC) agar containing streptomycin (100 μg/mL) and potassium tellurite (2.5 μg/mL) were used to suppress the normal flora and detect sorbitol-nonfermenting challenge O157 strain after overnight incubation at 37°C. Fecal and RAMS samples collected before the O157 challenge were plated as described above and also on SMAC agar containing only potassium tellurite to detect any naturally present O157 before oral challenge. Latex agglutination was used to serologically determine if sorbitol-nonfermenting colonies were O157 (*E. coli* O157 latex, Oxoid Diagnostic Reagents, Oxoid Ltd., Hampshire, United Kingdom). Detection sensitivity for direct plating (without enrichment) was ∼10^3^ colony-forming units (CFU) per 50 ml of sample suspension. For enrichment plating, samples were either assigned a value of one-log CFU or a value of 0 if these produced or did not produce O157-sepcific colonies, respectively. Total CFU per 200 mg sample was determined to maintain uniformity when comparing RAMS and fecal samples.

### 16S rRNA Gene Sequencing

Taxonomic profiles and microbiota diversity of the fecal and RAMS samples collected from the four Holstein steers in the pilot study were analyzed to determine differences between the two sample types as previously described (Mir et al., 2019). Similar analysis was done in the main study with the fecal and RAMS samples collected from all eight Jersey steers at nine time points resulting in a total of 144 samples for DNA extraction. DNA was extracted from 0.25 g fecal or RAMS (four swabs per animal rinsed in PBS and rinsate centrifuged to collect 0.25 *g* of mucosal material) samples using standard instructions provided with the DNeasy PowerSoil kit (Qiagen, Germantown, MD, United States). DNA yield and purity were evaluated using the Nanodrop (Life Technologies Corp., Grand Island, NY, United States) and verified by agarose gel electrophoresis. Previously described primers and PCR conditions were used to amplify and sequence the V4 region of the bacterial 16S rRNA gene (Kozich et al., 2013). The PCR mixture contained 17 μl of *Accu*Prime *Pfx* SuperMix (Life Technologies Corp., Grand Island, NY, United States), 5.0 μM of each primer, and 25 ng of the template DNA. PCR settings included denaturation at 95°C for 2 min and 22 cycles of amplification (20 s at 95°C, 15 s at 55°C, 5 min 72°C) followed by a final extension at 72°C for 10 min. PCR amplicons were normalized using the SequalPrep^TM^ Normalization Plate (96) Kit (Applied Biosystems Inc., Foster City, CA, United States). Normalized amplicons were pooled and quantified using Kapa SYBR Fast qPCR (Kapa Biosystems, Wilmington, MA, United States) and sequenced on a MiSeq Instrument using a MiSeq Reagent Kit v2 (Illumina, San Diego, CA, United States) following manufacturer’s instructions. DNA from a mock community with defined composition (Allen et al., 2016) and no template control (NTC, water) were also used to calculate sequencing error rates.

### Data Analysis

The sequences were analyzed in the Microbial Genomics Module (MGM) 1.6.1 (CLC Genomics Workbench, Qiagen Inc., Redwood City, CA, United States) following the manufacturer’s protocol for clustering of OTUs and as previously described (Mir et al., 2019). Specifically, the paired read data (forward and reverse sequences) were merged to create the highest quality sequences for clustering. The alignment parameters were set as one for mismatch cost, 40 for minimum score, four for gap cost, and five as the maximum unaligned end mismatches. Then the sequences were trimmed to a fixed length of 250 bp. Samples containing lower than 100 or less than 10% of the median number of sequences were filtered out before using the sequences in OTU clustering. The OTUs were clustered at 97% similarity against the SILVA 16S rRNA small subunit reference database version 128 (Quast et al., 2013) and the metadata were added to the abundance table to aggregate samples based on metadata attributes.

The curated sequences were submitted to the National Center for Biotechnology Information-GenBank database (Accession # PRJNA598032, ID # 598032) and aligned in the MGM module using MUSCLE by the neighbor joining method and following the Jukes-Cantor model (CLC Genomics Workbench, Qiagen) as previously described (Mir et al., 2019). This alignment was used to create a maximum likelihood phylogenetic tree. The phylogenetic tree and the OTU table describing the taxonomic differences among RAMS and fecal samples, and between Chx and Mck groups, were used to calculate the Bray-Curtis dissimilarity and generate the PCoA plot. The differences in beta diversity between groups was analyzed using the Permutation Multivariate Analysis of Variance (PERMANOVA) in the MGM module (CLC Genomics Workbench, Qiagen). This distance-based method tests the association of microbiome composition with any covariates of interest. The analysis and comparisons of alpha diversity (represented here by measuring Shannon diversity index and Chao 1 species richness) between groups was carried out after OTU tables were rarefied to the sample containing the lowest number of sequences (subsampled to 1580 sequences per sample). Alpha diversity measures were calculated in the MGM 1.6.1 (CLC Genomics Workbench, Qiagen) and compared between fecal and RAMS samples, using the Student’s *T*-test. The difference in alpha diversity among two groups of steers (challenged and mock) at Days 1, 8, 15, 22, and 29 after the O157 challenge, was analyzed by one-way ANOVA and a cutoff value of 0.05 (*p* < 0.05) used to determine statistical significance using GraphPad Prism^®^ (Version 7.0c). To determine the taxa that significantly differ between groups, we used differential abundance analysis (DAA) with the OTU table (at genera-level, taxon = Genus) as input data. *Post-hoc* test (the false discovery rate, FDR) was used to determine significantly different (FDR *p*-value < 0.05) taxa between groups.

### Quantitative PCR (qPCR) and Data Analysis

Singleplex (containing one primer pair) qPCR assays were performed in a total volume of 20 μl, which was made up of 10 μl of 2x iTaq Universal SYBR Green Supermix (Bio-Rad, Hercules, CA, United States), 1 μl (0.4 μM) each of forward and reverse primers, 1 μl DNA (10 ng), and 7 μl of PCR-grade water. DNA was prepared from the fecal or RAMS samples as described above in the 16S rRNA gene sequencing method. 10 ng DNA per group used in the PCR was prepared by pooling 2.5 ng DNA derived from samples representing each of the four animals in the RAMS-Chx, RAMS-Mck, fecal-Chx, and fecal-Mck groups (Figure 1A). Previously published primer pairs, two of which were designed for specific amplification of 16S rRNA gene sequences in *Firmicutes* and *Bacteroidetes*, and a third universal primer set targeting all bacterial 16S rRNA gene sequences, were used (Supplementary Table S2; Koliada et al., 2017). The qPCR reactions were set up in triplicate and run in 96-well plates on the CFX96 real-time PCR detection system (Bio-Rad, Hercules, CA, United States). The PCR system was programed to run for 5 min at 95°C (one cycle); 95°C for 10 s, 55°C for 30 s (data collection), 72°C for 30 s (35 cycles); and a melting curve segment. The relative abundance of *Firmicutes* and *Bacteroidetes* was computed by the ΔCt method and expressed as a ratio of *Firmicutes or Bacteroidetes* to total bacterial abundance. These ratios were calculated by the equation 2^Ct (total bacterial population) – Ct (*Firmicutes* or *Bacteroidetes*). Statistical significance was determined using the Holm-Sidak method, with alpha = 0.05; computations assumed that all rows are sample from populations with the same scatter (SD).

## Results

### Bacterial Composition of the RAJ Mucosa Differed From That of Feces Among Cattle in the Pilot and Main Study

The PERMANOVA analysis of the microbiota data from four fecal and four RAMS samples that were collected from healthy, non-challenged Holstein steers in the pilot study indicated a significant difference in the bacterial community structure of feces compared to RAMS (*p*-value < 0.05). This difference in bacterial diversity can be visualized in the principal coordinate analysis shown in Supplementary Figure S1. The higher the distance between spheres in Supplementary Figure S1, the greater is the variation in the microbial diversity between samples. Microbiome analysis of four donor cattle indicated that the difference in microbial diversity between fecal and RAMS samples is significant. The PERMANOVA analysis on the beta-diversity between fecal and RAMS showed a *p-*value (Bonferroni corrected) of < 0.001 (Supplementary Table S1). DAA of the taxonomic profiles indicated higher abundance of genera like *Anaeroplasma* and *Ruminococcaceae UCG-010* in feces while genera like *Corynebacterium* and *Bifidobacterium* were abundant in RAMS samples (Supplementary Figure S2). These results verified inherent differences in the fecal and RAMS microbiota besides validating that the sampling technique maintained the differences between the two samples. Using the same sampling techniques, distinctively abundant genera were also observed in the fecal and RAMS microbiota of the Jersey steers used in the main study where irrespective of O157 colonization fecal samples had higher relative abundance of *Ruminococcus* while *Corynebacterium* was relatively more abundant in RAMS samples as described below.

### O157 Was Isolated Only From the Challenged (Chx) Group of Steers in the Main Study

No adverse clinical signs or health issues were observed among the animals after oral challenge and throughout the course of this study. Fecal and RAMS samples were negative for O157 among the non-challenged cattle used in the pilot study and the mock PBS-challenged (Mck) group of cattle in the main study (Figure 1B). In the latter study, culture results were negative for the presence of any natural O157 in fecal and RAMS samples collected from steers in the Chx group, before oral administration of the challenge O157 strain. After oral inoculation, all four challenged steers shed the challenge O157 strain in feces and RAMS samples till the end of study (day 29), although the average concentration (CFU/200 mg sample) was lower in RAMS samples compared to the fecal samples (Figure 1B). Significant difference in O157 counts between fecal and RAMS samples was observed on days 1 (*p-*value = 0.0036), 3 (*p-*value = 0.0007), 6 (*p-*value = 0.0324), 8 (*p-*value < 0.0001), and 15 (*p-*value = 0.0197) (Figure 1B).

### O157 Colonization Influenced Microbial Community Structure of Fecal and RAJ Mucosa Samples in the Main Study

**FIGURE 2:**
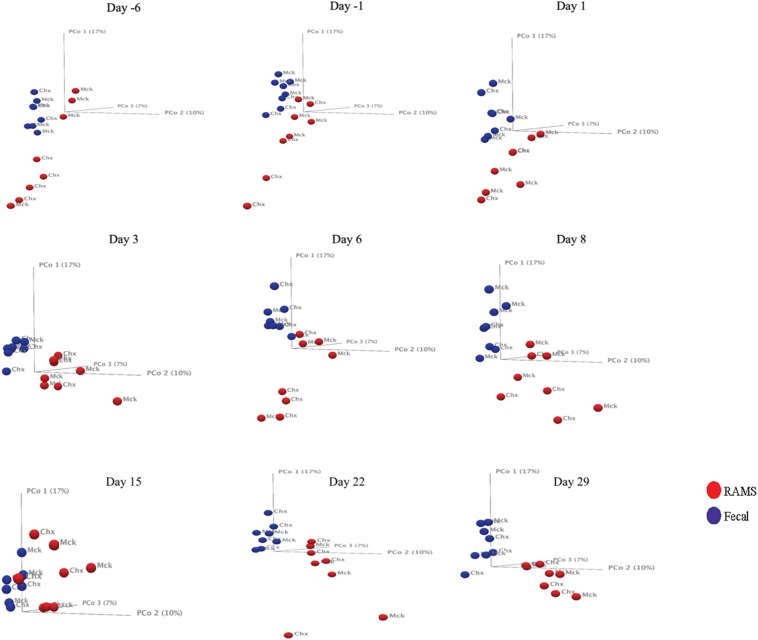
Principal Coordinate Analysis (PCoA, beta-diversity) plots comparing microbial diversity between fecal (blue spheres) and RAMS (red spheres) samples over sampling days -6, -1, 1, 8, 15, 22, and 29. The spheres are further labeled according to the two treatment groups: O157 challenged (Chx) and mock PBS/non-challenged (Mck). Significant differences in microbial diversity between fecal and RAMS samples were observed before and after oral challenge with O157. However, for RAMS samples, the spheres were further apart from each other after O157 challenge indicating increased beta diversity between fecal and RAMS samples.

**FIGURE 3:**
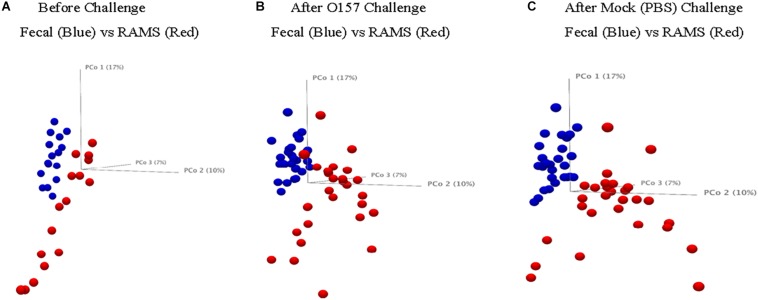
Principal Coordinate Analysis (PCoA, beta-diversity) plots comparing microbial diversity between fecal (blue spheres) and RAMS (red spheres) samples from animals in the Chx group **(A)** before O157 challenge, **(B)** after O157 challenge, and **(C)** from animals in the Mck group after PBS challenge. The samples were pooled based on the colonization/challenge status to determine association between O157 shedding and microbial diversity. RAMS samples (red spheres) were further apart from each other after O157 challenge indicating increased beta diversity between fecal and RAMS samples.

The 16S rRNA gene sequencing resulted in 5.02 million reads from 144 samples which, after filtering and removing chimeras, yielded 3.66 million reads that were used to create a table of OTUs. OTUs with fewer than 10 reads across the samples were removed to create a filtered OTU table and sequences were matched to reference database to yield a genus level OTU table containing 354 bacterial genera. This OTU table and the sample information (metadata) were used to determine statistical differences in bacterial community structure, as previously described (Mir et al., 2019). There was a significant difference (*p*-value < 0.05) in the bacterial community structure between O157-shedding (Chx) and non-shedding (Mck) animal samples (both fecal and RAMS) (Supplementary Table S1 and Figure 2). Also, the overall bacterial community structure was different between fecal and RAMS samples before and after challenge (O157/PBS) regardless of O157 shedding status (*p*-value < 0.05) (Supplementary Table S1 and Figures 2, 3). However, for RAMS samples, the spheres were further apart from each other after O157 challenge indicating increased beta diversity between fecal and RAMS samples (Figures 2, 3).

**FIGURE 4:**
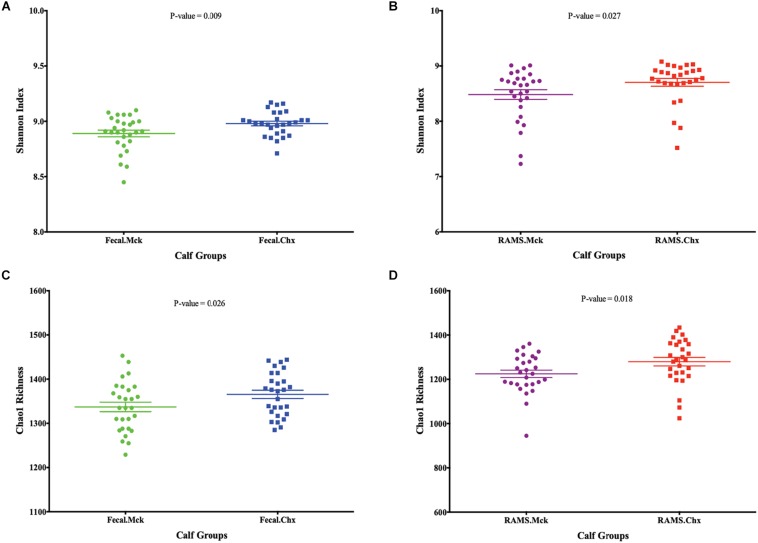
Post-challenge (Mck or Chx) alpha-diversity represented as scatter plots of Shannon Index and Chao 1 species richness estimates (Mean ± SEM) for **(A,C)** fecal and **(B,D)** RAMS samples, respectively. All *p*-values were obtained using the unpaired one-tailed *T*-Test (*p* < 0.05).

The analysis and comparisons of alpha-diversity (represented by measuring Shannon diversity index and Chao 1 species richness) between groups was carried out after subsampling all the samples to 1580 sequences per sample (rarefaction). The Chao 1 species richness and Shannon diversity index were higher in samples from animals in the Chx group that were positive for O157 compared to the O157-negative samples from Mck controls after mock challenge with PBS (Figure 4). The results obtained with Shannon index and species richness correlated with the dynamic changes seen in community structure (Supplementary Table S1 and Figure 2) wherein the RAMS samples showed a marked change in microbiome after oral challenge.

### O157 Challenge Affected the *Firmicutes*: *Bacteroidetes* (F:B) Ratio but Not *Proteobacteria* Abundance at the RAJ Mucosa and Feces

**FIGURE 5:**
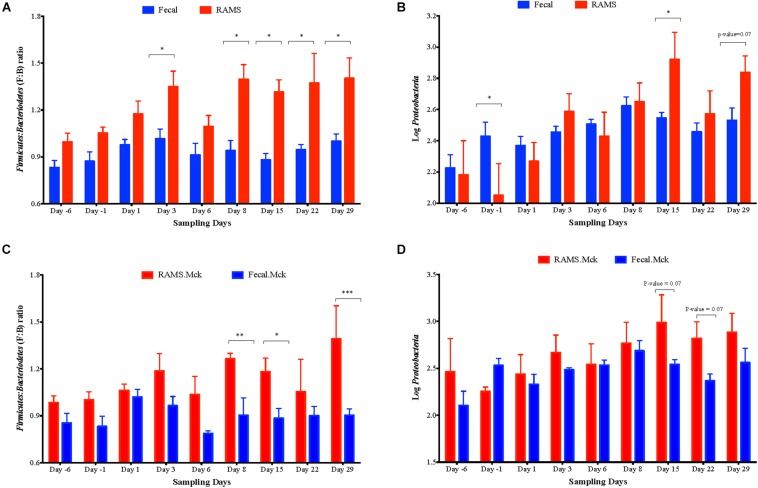
Overall *Firmicutes: Bacteroidetes* (F: B) ratio **(A)** and the relative abundance of *Proteobacteria* **(B)** measured as Log_10_ of number of sequences between pooled fecal (blue bars) and RAMS (red bars) samples, at Days -6, -1, 1, 3, 6, 8, 15, 22, and 29. Same data derived from RAMS and fecal samples of mock (PBS)-challenged animals is shown in **(C,D)** as comparative controls. Significant F:B ratio differences were observed between fecal and RAMS samples after Day 1. *Proteobacterial* abundance was significantly different at Day -1, Day 15 and Day 22 of sampling. Data is shown as mean ± SEM for both F: B ratio and abundance of *Proteobacteria* at each sampling point. All *p-*values were obtained using the unpaired one-tailed *T*-Test (*p* < 0.05). *p < 0.05, **p < 0.01, and ***p < 0.001.

**FIGURE 6:**
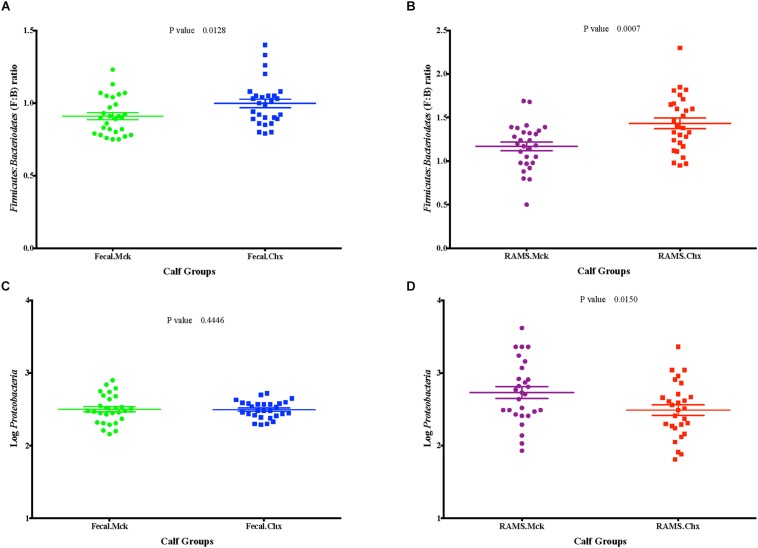
Post-challenge (Mck or Chx) **(A,B)** *Firmicutes: Bacteroidetes* (F: B) ratio and **(C,D)** the relative abundance of *Proteobacteria* (measured as Log_10_ of number of sequences) between fecal and RAMS samples.

Overall the F:B ratio was higher for the RAMS samples compared to the fecal samples and this difference was significant post-O157 challenge at Days 3, 8, 15, 22, 29 (*p*-value < 0.05; Figure 5), based on the 16S rRNA gene sequencing. Specifically, the F:B ratio was higher in RAMS from Chx (O157 challenge) versus RAMS from Mck (PBS challenge) steers (*p*-value = 0.0007; Figure 6) and this difference was especially significant at Day 22 (*p*-value < 0.05) between the Chx and Mck groups (Supplementary Figure S3).

No such trend was observed for the overall abundance of the phylum *Proteobacteria* (measured as Log_10_ of number of OTUs) between the fecal and RAMS samples post-O157 challenge except on day 15 (*p*-value < 0.05) and day 29 (*p*-value = 0.07) (Figure 5). However, even this difference in *Proteobacteria* abundance was not due to O157 challenge; *Proteobacterial* abundance was consistently lower in RAMS samples from the Chx versus Mck animals, pre- and post-O157 challenge (Figure 6 and Supplementary Figure S3).

A higher F:B ratio was also observed in fecal samples from the Chx versus Mck groups (*p*-value 0.0128; Figure 6). Analysis of individual sampling points, pre-and post-O157 challenge, showed significant F:B ratio difference at Day 6 between fecal samples from the Chx versus Mck groups (*p*-value < 0.05; Supplementary Figure S4). However, *Proteobacteria* abundance did not differ between fecal samples collected post-challenge from Chx and Mck animal groups (Figure 6) overall, or at any specific collection time points (Supplementary Figure S4).

The relative abundance of the two major phyla *Firmicutes* and *Bacteroidetes* was further determined by qPCR for samples collected on Days 1, 6, and 22. The RAMS-Chx and RAMS-Mck samples collected on Day 22, and the fecal-Chx and fecal-Mck samples collected on Day 6, had significant difference in the F:B ratios based on 16S rRNA gene sequencing (Supplementary Figures S3, S4); Day 1 was selected for comparative purposes. As shown in Supplementary Figure S5, relative abundance for *Firmicutes* was higher for RAMS compared to fecal samples and higher for Chx samples within each sample type compared to the Mck samples. This difference was particularly significant at Day 22 for RAMS-Chx versus fecal-Chx (*p* = 0.001852) (Supplementary Figure S5A), and RAMS-Chx versus RAMS-Mck (*p* = 0.016936) (Supplementary Figure S5C) samples. These results were comparable to that observed with 16S rRNA sequencing verifying those results. Interestingly, contrary to the 16S rRNA sequencing results, the qPCR results for Day 6, fecal-Chx versus fecal-Mck samples only approached significance (*p* = 0.072823) reflecting the specificity of this targeted quantitative assay.

### Distinct Genera in the RAJ Mucosal Microbiota Could Be Associated With O157 Colonization

The sequencing data was used to create the taxonomic profiles of GIT microbiota of all steers corresponding to all sampling points (before and after O157 or PBS challenge), as previously described (Mir et al., 2019). The fecal and RAMS samples collected after O157 challenge indicated significant differences in community structure between Chx and Mck groups (Pseudo-f statistic 2.91, *p*-value < 0.01; Supplementary Table S1). We next performed DAA on OTU at the genera level to determine which members of the bacterial community were associated with the significant changes in fecal and RAMS microbiota due to oral challenge with O157. To get the useful information, we selected bacterial genera with overall abundance more than 0.1% to avoid sequencing errors, and any genera for which taxonomy was not available.

The DAA analysis at genera level was more informative compared to the family level because certain members of one bacterial family could be differentially abundant in two groups. For example, *Lachnospiraceae FCS020 group* was relatively more abundant in RAMS samples at Days 3, 6, 8, 15, and 22 while *Lachnospiraceae NK4A136 group* was relatively more abundant in fecal samples at day 8 of sampling (Supplementary Figure S6). On the other hand, results of DAA analysis on OTU table at genera level were in accordance with the observations made about bacterial community structure and indicated that higher number of bacterial genera were differentially abundant between fecal and RAMS samples after O157 challenge (Supplementary Figure S6).

**FIGURE 7:**
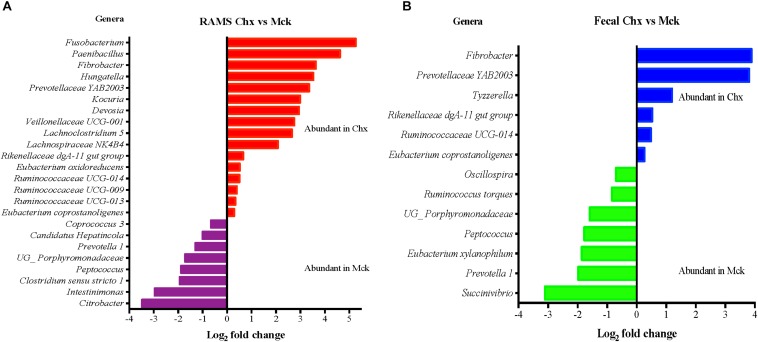
Differential abundance analysis (DAA) of OTUs (genus-level) between RAMS and fecal samples based on their shedding status after O157 (Chx) or mock (Mck) challenge is shown for RAMS **(A)** and fecal **(B)** samples. OTU tables were rarefied to the sample containing the lowest number of sequences in each analysis. OTUs were assigned at the genus level, and genera with relative abundance of more than 0.1% of the total were used in DAA analysis. For multiple comparisons, an estimate of the false discovery rate (FDR) was calculated to determine differentially abundant genera with a significance threshold of *p* < 0.05. The genera represented by the red and blue bars are relatively more abundant in the RAMS and fecal samples of the Chx group. The genera represented by the purple and green bars are relatively more abundant in the RAMS and fecal samples of the Mck group.

Overall fecal samples had higher relative abundance of *Ruminococcus* while *Corynebacterium* was relatively more abundant in RAMS samples (Supplementary Figure S6). In addition, DAA analysis on the OTU table at genera level indicated higher relative abundance of *Fusobacterium* and *Paenibacillus* in RAMS samples from O157 shedding cattle in the Chx group while *Intestinimonas* and *Citrobacter* were abundant in the RAMS samples from Mck animals (Figure 7). DAA analysis of fecal samples indicated *Tyzzerella* was uniquely abundant in O157-shedding cattle in the Chx group while *Succinivibrio*, and *Prevotella 1* were uniquely more abundant in fecal samples from cattle in the Mck group (Figure 7).

## Discussion

Gastrointestinal tract microbiota plays a significant role in the development and regulation of the immune response to bacteria, and an altered GIT microbiota (dysbiosis) can affect outcome of bacterial infection/colonization (Oh et al., 2014; Nguyen et al., 2016). Knowledge of GIT microbiota of domestic animals, such as cattle, is limited as evidenced by the relatively small number of studies compared to the ones evaluating the human microbiome (Callaway et al., 2010; Li et al., 2017). One such study identified OTUs from genera like *Alistipes, Blautia, Oscillospira*, and *Prevotella* to be more abundant in O157 super-shedding cattle while two OTUs representing *Proteobacteria* and *Tenericutes* were more abundant in non-shedding cattle (Xu et al., 2014). Another study reported higher abundance of genera like *Prevotella* and *Treponema* in non-shedders, while *Ruminococcus*, and *Selenomonas* were more abundant in O157 super-shedders when microbiota from GIT tissues and contents collected at slaughter were analyzed (Zaheer et al., 2017). In another study, *Janibacter, Paraprevotella*, and *Pedobacter* were relatively more abundant in RAJ tissues from non-shedding cattle while genera like *Corynebacterium, Gordonia*, and *Luteococcus* were more abundant in RAJ tissues from super-shedding cattle (Wang et al., 2018).

Given that most of the GIT microbiota studies in cattle are field studies and performed in the context of the O157 super-shedding phenomenon we sought to evaluate changes in the microbiota relative to O157 colonization under controlled experimental conditions. We specifically analyzed microbiota composition and perturbations at the RAJ mucosa in comparison to feces. Our screen of O157-negative Holstein steers, in the pilot study, gave insights into the distinct microbiota composition of the RAJ similar to previous reports (Zaheer et al., 2017; Wang et al., 2018) with a higher abundance of *Corynebacterium* in RAMS samples compared to the abundance of *Ruminococcaceae* in the feces of these animals. These distinctively abundant genera were also observed in the RAJ and fecal microbiota of the Jersey steers, used in the main study, despite the difference in animal breed and irrespective of O157 colonization.

O157 colonization was also associated with changes in the *Firmicutes*: *Bacteroides* (F:B) ratio of both feces and RAMS samples. The three dominant phyla that comprise the GIT microbiota in cattle, goats, and most mammals are *Bacteroidetes, Firmicutes*, and *Proteobacteria* (Lee et al., 2012; Jami et al., 2014; Malmuthuge et al., 2014). These three bacterial phyla and certain genera comprising these phyla including, *Faecalibacterium* and *Ruminococcus* (*Firmicutes*), *Prevotella*, and *Bacteroides* (*Bacteroidetes*), and *Succinovibrio* (*Proteobacteria*) have been consistently observed in bovine feces and are suggested to be a part of the bovine resident microbiota (Durso et al., 2010). Bovine fecal microbiota was found to be dominated by the *Firmicutes* (relative abundance: 53.9%), *Bacteroidetes* (35.6%), and *Proteobacteria* (1.7%) phyla in O157 super-shedder and non-shedder cattle (Xu et al., 2014). Likewise, in GIT samples collected at the time of slaughter, the *Firmicutes* and *Bacteroidetes* phyla comprised more than 70% of all sequences and relative abundance of *Proteobacteria* ranged from as high as 16% in the duodenum of O157 super-shedding cattle to as low as 0.5% in the distal jejunum of non-shedding cattle (Zaheer et al., 2017). The *Firmicutes* (61.5%), *Bacteroidetes* (27.9%), and *Proteobacteria* (5.5%) were also the most abundant phyla in the rectoanal junction mucosa-associated microbiota (Wang et al., 2018). A reduction in the abundance of *Firmicutes* and increase in the abundance of *Bacteroidetes* and *Proteobacteria* have been regarded as an indicator of GIT microbiota dysbiosis (Sampson et al., 2016; Li et al., 2017). Increased relative abundance of *Firmicutes* has been associated with higher levels of short-chain fatty acids (SCFAs) (Turnbaugh et al., 2006) due to an increased efficiency in energy extraction from the diet and short-chain fatty acids, including butyrate and propionate, which have been suggested to decrease the shedding of O157 in cattle (Turnbaugh et al., 2006; Jacob et al., 2009).

In this study, we observed a higher F:B ratio in fecal and RAMS samples of animals colonized with O157 using two different methods of 16S rRNA sequencing and qPCR; overall this ratio was significantly higher for RAMS samples post-O157 challenge. Although this increase in the F:B ratio did not result in clearance of O157, the increased relative abundance of *Firmicutes* may have contributed to the lower concentration of O157 recovered from the RAJ mucosa of O157-shedding animals. On the other hand, O157 shedding had no influence on *Proteobacteria* abundance suggesting minimal dysbiosis, which was in agreement with a previous study (Xu et al., 2014). Taxonomic profiles of fecal and RAJ microbiota associated with changes in the community structure due to O157 colonization were also analyzed. Most of the changes causing bacterial diversity shifts could be attributed to different bacterial genera of the same family (ex. *Lachnospiraceae*) or re-distribution/enrichment of specific genera. For instance, *Campylobacter* was abundant at Day 1 in RAMS from non-shedding cattle but found to be relatively abundant at Day 6 in RAMS from O157 shedding cattle. However, using DAA we also identified differentially abundant bacterial genera, specific to the RAMS samples, associated with O157 colonization (*Fusobacterium* and *Paenibacillus*) which would be useful in future studies identifying a causal relationship. Mock-challenged cattle showed higher relative abundance of *Intestinimonas* and *Citrobacter* in RAMS samples. Thus, the bacterial community change was associated with the sample type which may have an influence on the site-specific colonization by O157 at the RAJ. Further studies are being planned to determine, (i) effects of different diets and challenge O157 strains on RAJ and fecal microbiota, (ii) alterations in the functional profile of the GIT microbiota and in major phyla due to O157 challenge specifically at the bovine RAJ, and (iii) microbiota changes over an extended period of time to see if any of these revert to pre-colonization status once O157 shedding by the challenged animals becomes non-detectable.

## Data Availability Statement

The raw sequencing data has been deposited with the National Center for Biotechnology Information-GenBank database (Accession # PRJNA598032 and ID # 598032).

## Ethics Statement

The animal study was reviewed and approved by the USDA-ARS-Institutional Animal Care and Use Committee, National Animal Disease Center, Ames, IA, United States.

## Author Contributions

IK, VS, TL, HA, RM, and RS developed the study concept and design, and critically revised the manuscript. RM, IK, and VS performed the experiments and analyzed the data. RM and IK drafted the manuscript. All authors have read and approved the manuscript.

## Disclaimer

Mention of trade names or commercial products in this article is solely for the purpose of providing specific information and does not imply recommendation or endorsement by the United States Department of Agriculture. USDA is an equal opportunity provider and employer.

## Conflict of Interest

The authors declare that the research was conducted in the absence of any commercial or financial relationships that could be construed as a potential conflict of interest.
