# Supplementary figures and images for: Recto-Anal Junction (RAJ) and Fecal Microbiomes of Cattle Experimentally Challenged With Escherichia coli O157:H7
Source: Front Microbiol. 2020 Apr 17;11:693. doi: 10.3389/fmicb.2020.00693 (PMC7181329; doi:10.3389/fmicb.2020.00693)

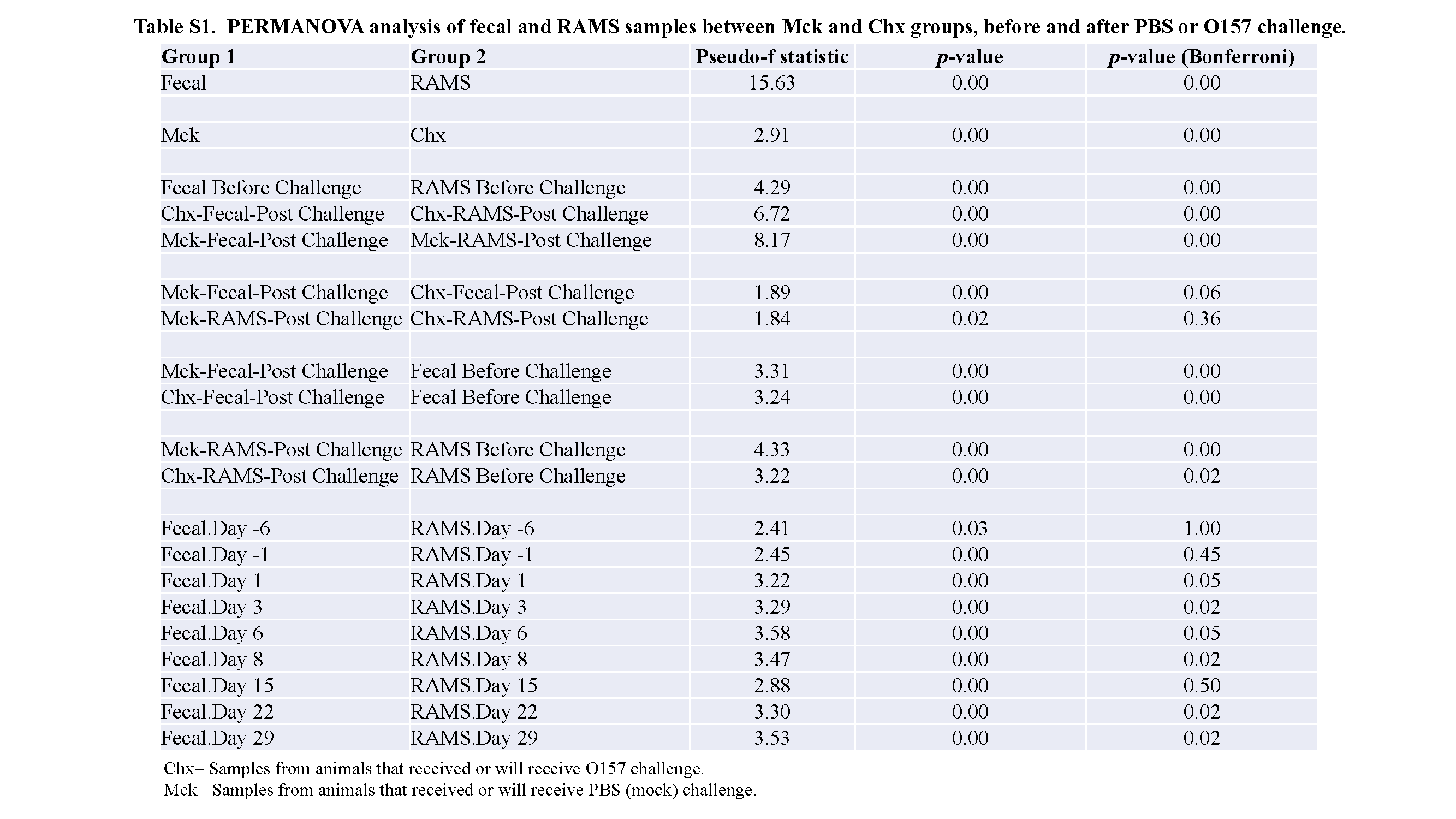

Supplement: Supplementary file 1 [file Data_Sheet_1.zip › Table S1.tiff]

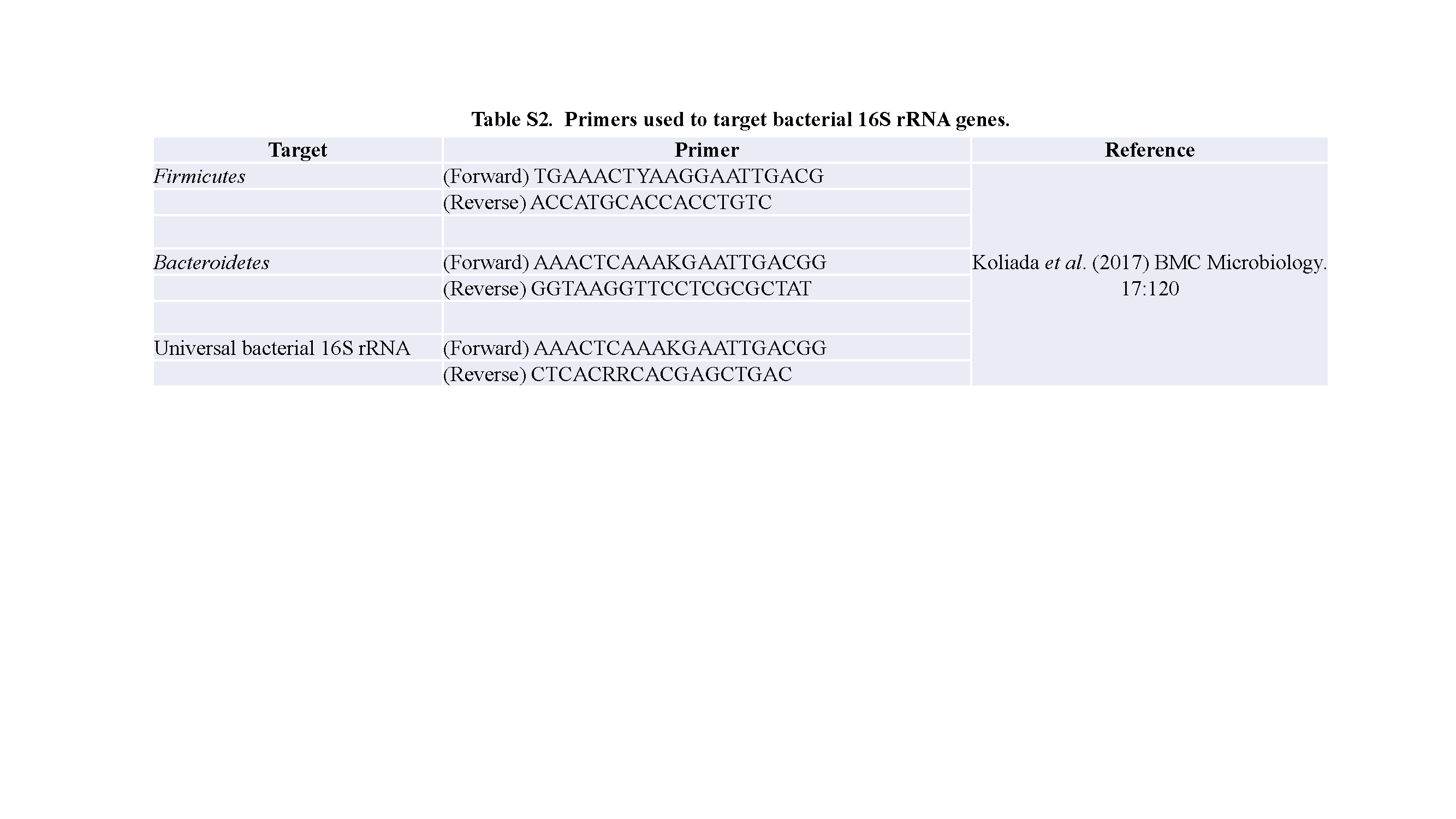

Supplement: Supplementary file 1 [file Data_Sheet_1.zip › Table S2.tiff]

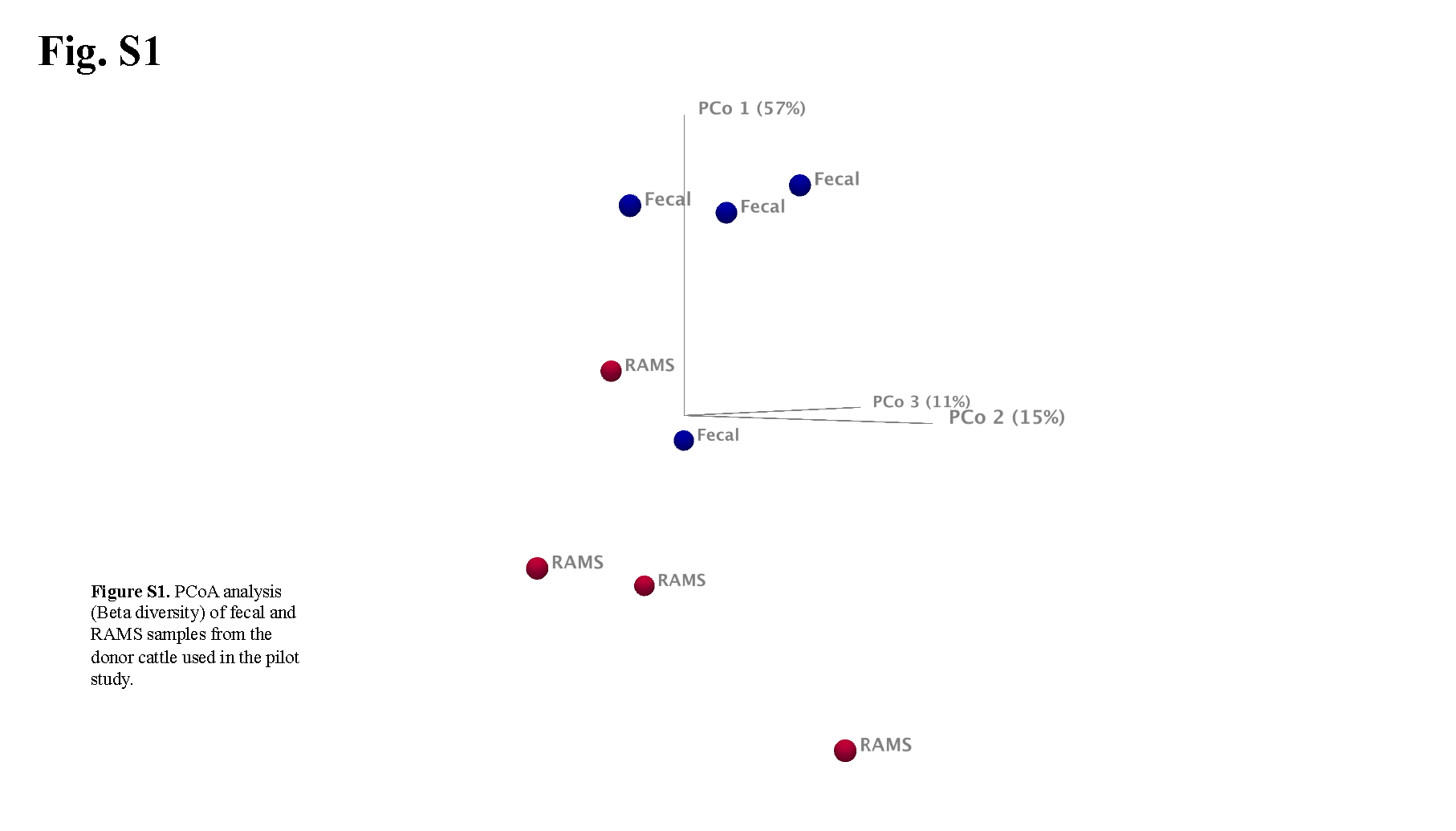

Supplement: Supplementary file 2 [file Data_Sheet_2.zip › Figure S1.tiff]

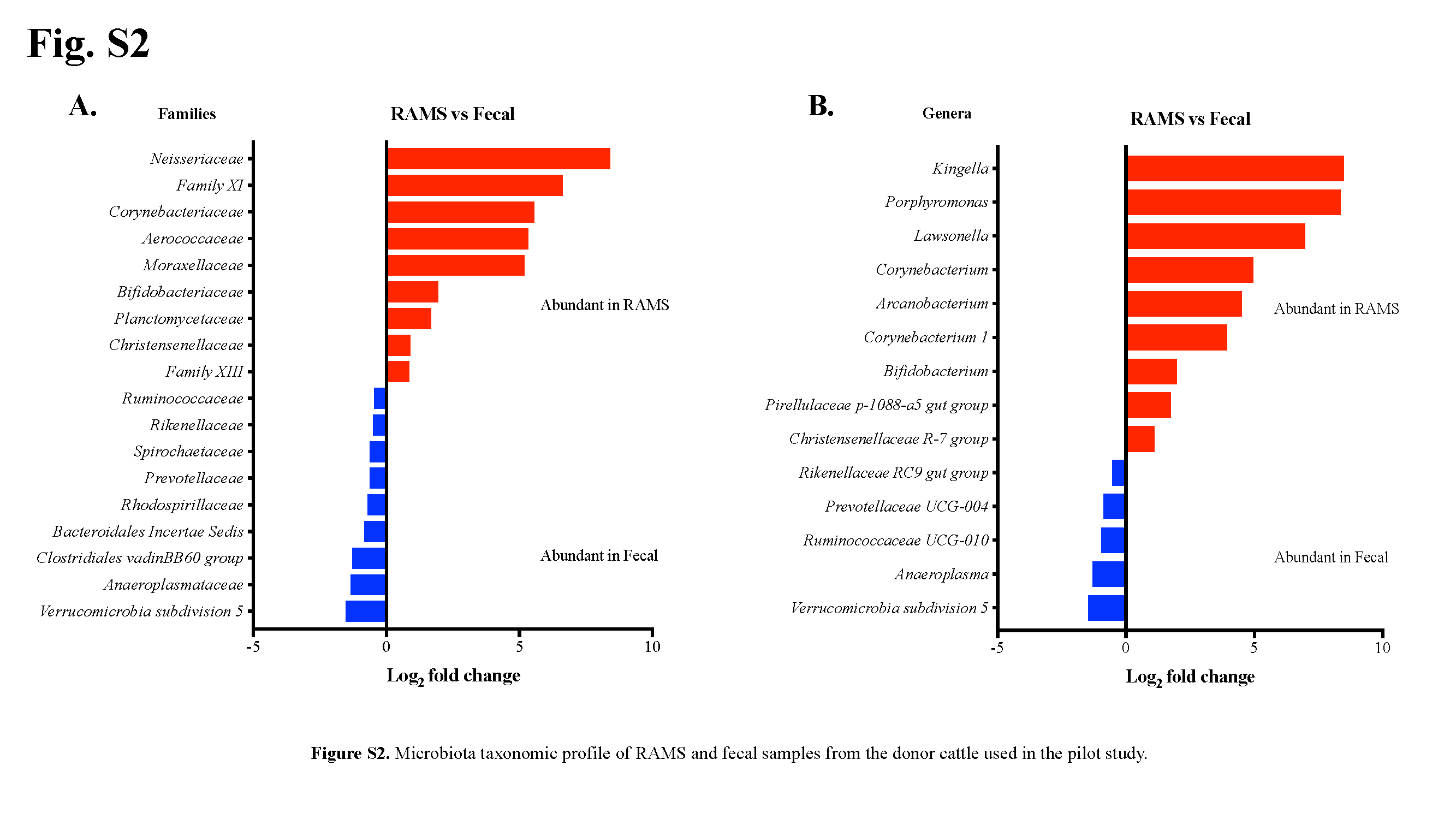

Supplement: Supplementary file 2 [file Data_Sheet_2.zip › Figure S2.tiff]

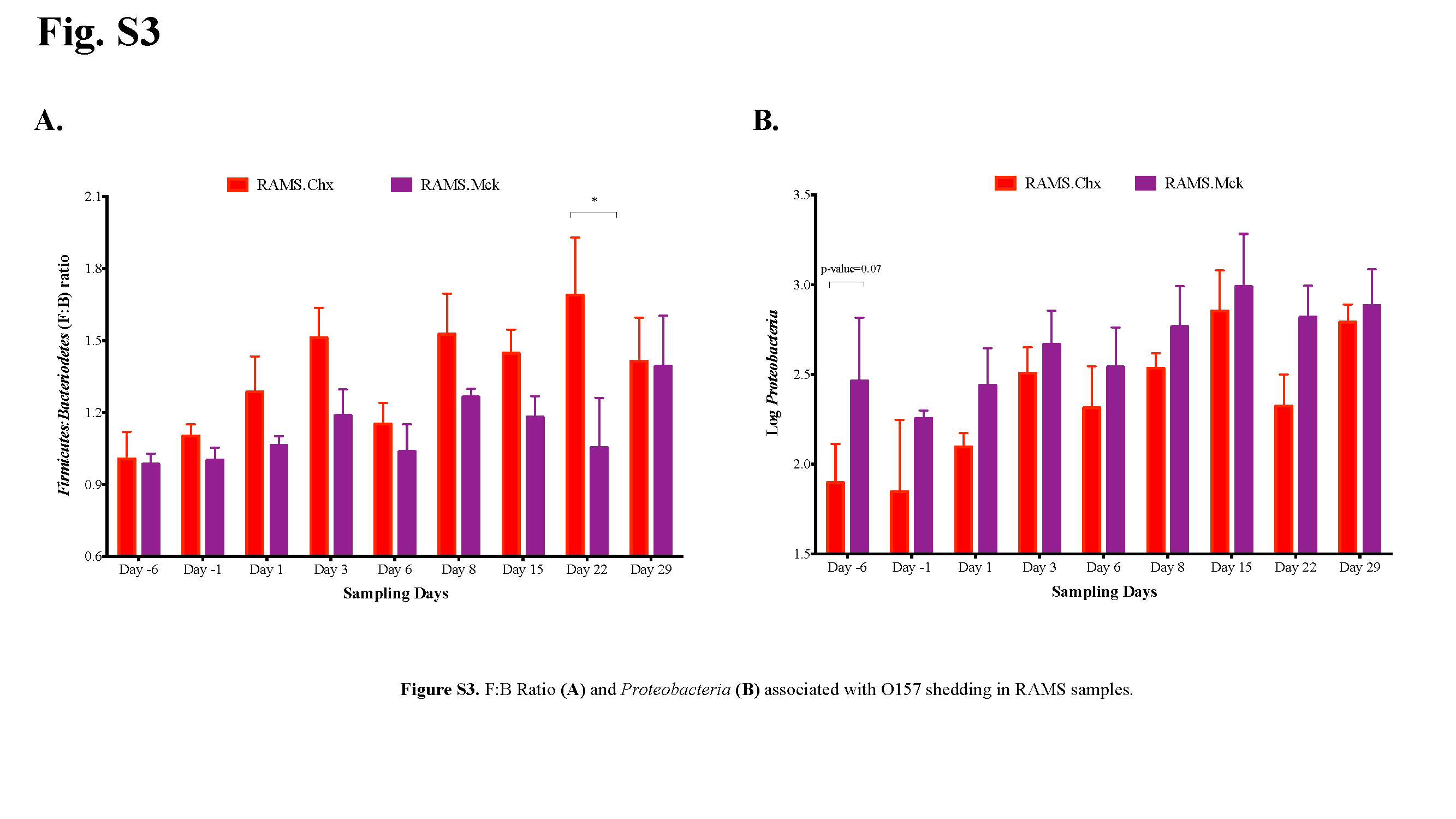

Supplement: Supplementary file 2 [file Data_Sheet_2.zip › Figure S3.tiff]

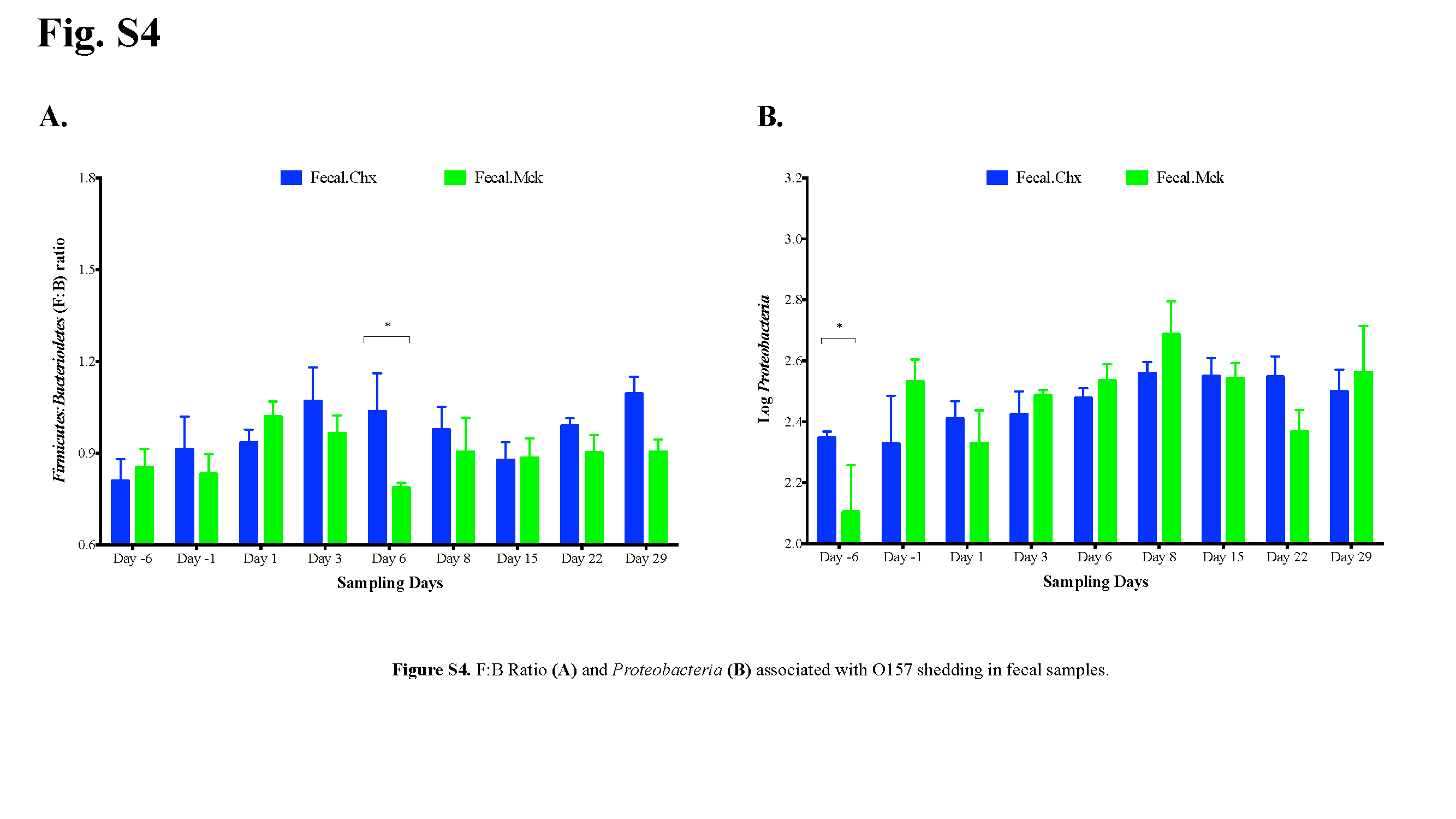

Supplement: Supplementary file 2 [file Data_Sheet_2.zip › Figure S4.tiff]

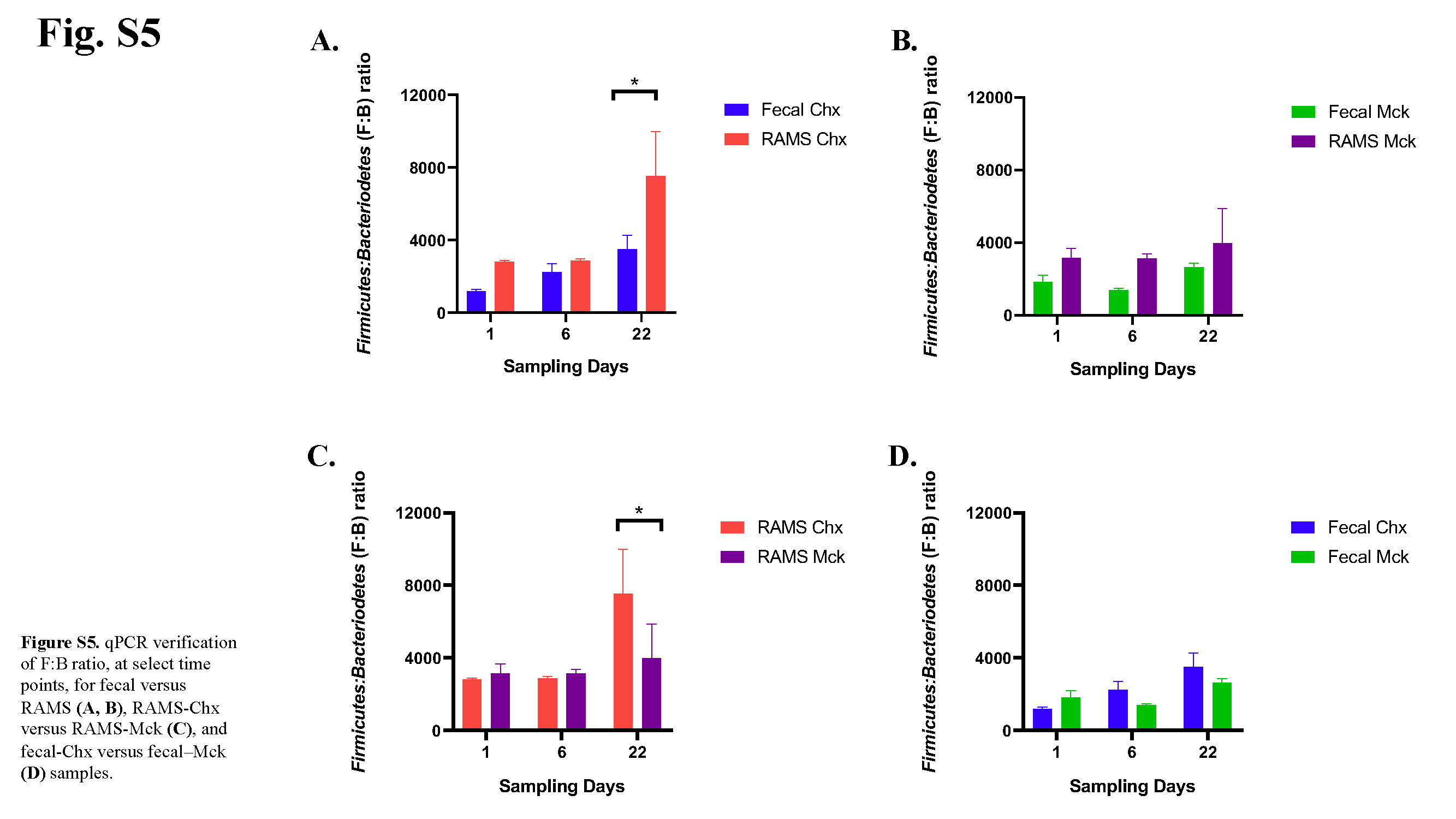

Supplement: Supplementary file 2 [file Data_Sheet_2.zip › Figure S5.tiff]

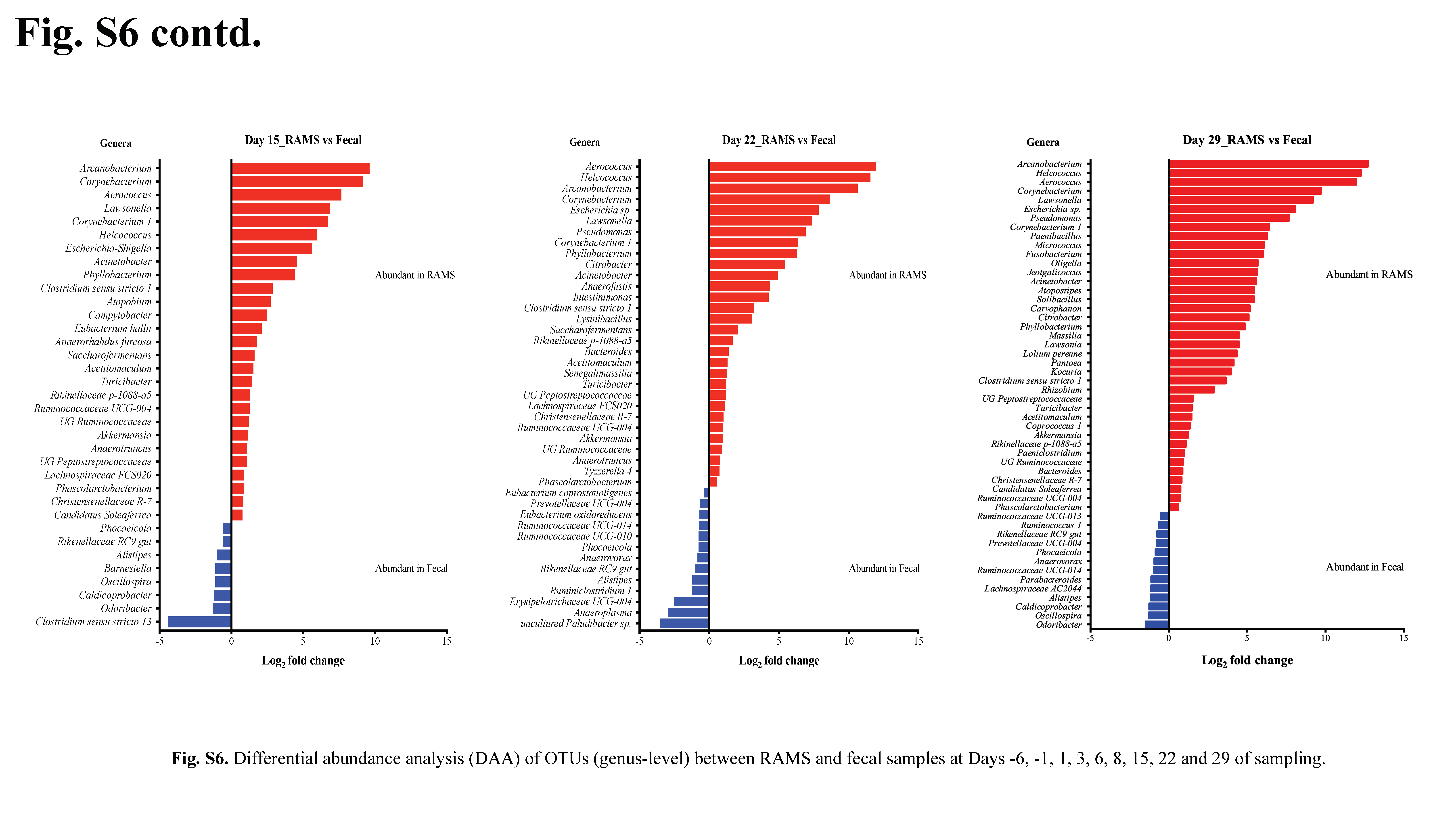

Supplement: Supplementary file 2 [file Data_Sheet_2.zip › Figure S6 cont.tiff]

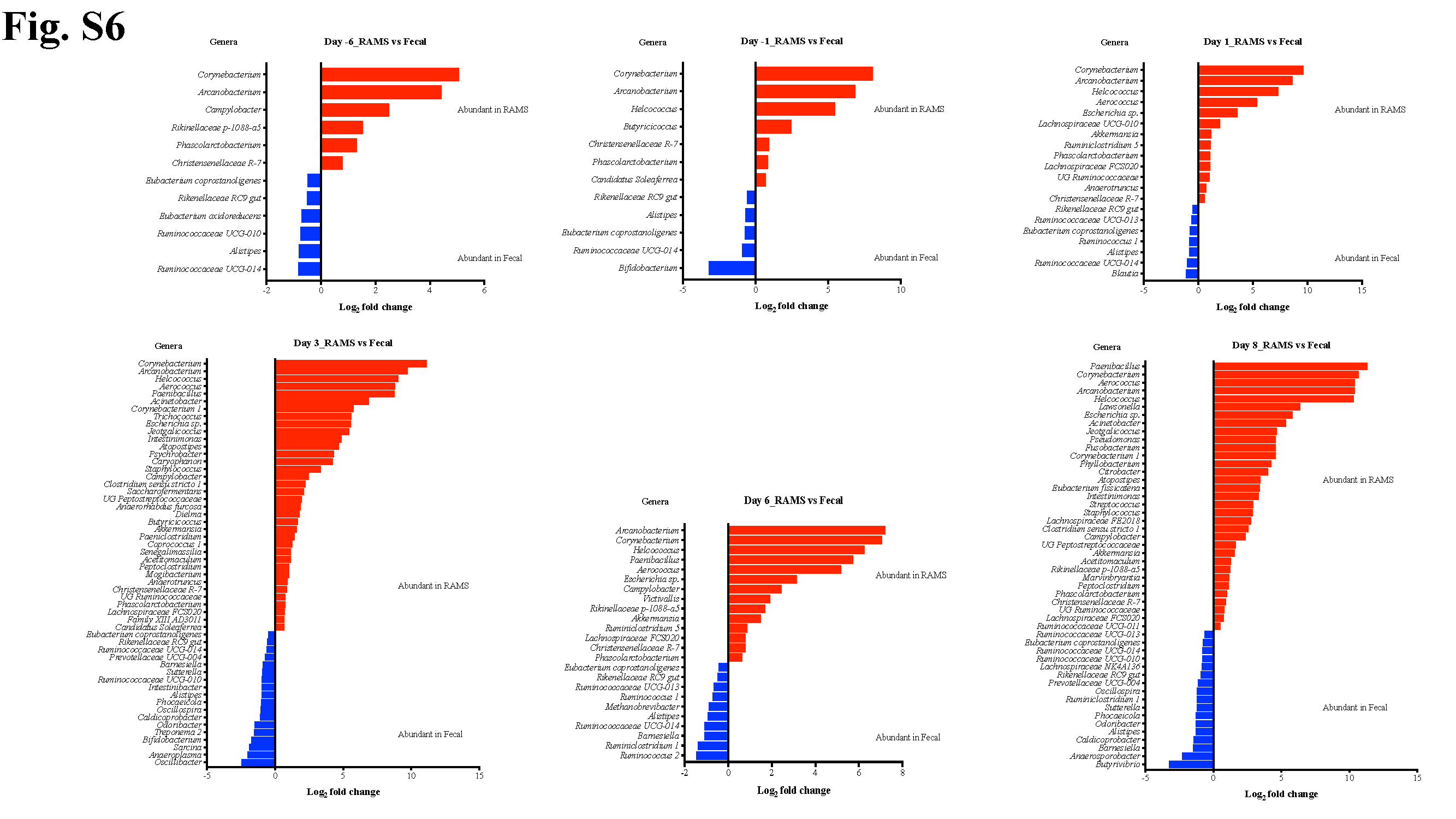

Supplement: Supplementary file 2 [file Data_Sheet_2.zip › Figure S6.tiff]
